# Aptian archaeoniscid isopod from the Araripe Basin supports Tethyan-linked dispersal into Gondwanan lacustrine systems

**DOI:** 10.1038/s41598-026-59890-0

**Published:** 2026-07-19

**Authors:** Daniel Lima, Arthur Anker, Sam W. Heads, Allysson P. Pinheiro, William Santana

**Affiliations:** 1 Museum of Paleontology Plácido Cidade Nuvens, Universidade Regional Do Cariri (URCA), Santana Do Cariri, Ceará, Brazil; 2 Biological and Environmental Science and Engineering Division, King Abdullah University of Science and Technology (KAUST), Thuwal, Saudi Arabia; 3 Illinois Center for Paleontology, University of Illinois Urbana-Champaign, Champaign, IL USA

**Keywords:** Malacostraca, Sphaeromatidea, Fossil isopod, Crustacean, Santana group, Archaeoniscidae, Crato formation, Evolution, Solid Earth sciences, Zoology

## Abstract

A new genus and species of fossil isopod, *Cicero spectabilis* n. gen. n. sp. is described from the Early Cretaceous (Aptian) Nova Olinda Member of the Crato Formation, Araripe Sedimentary Basin, Ceará, northeastern Brazil. The holotype, preserved in laminated limestones, represents the first confirmed record of the family Archaeoniscidae within this stratigraphic unit and extends the previously known paleogeographic distribution of archaeoniscid-like isopods into Gondwana lacustrine settings. The new genus is distinguished from other taxa currently referred to Archaeoniscidae—*Archaeoniscus*, *Ferreniscus* and *Codoisopus*, by its broad, ovate body, distinctive antennular subdivision, and a fan-like caudal structure formed by birramous uropods with well-differentiated exopod and endopod, combined with a rounded subrectangular pleotelson bearing a row of posterior setae. The exceptional preservation of *C. spectabilis* n. gen. n. sp. enables a detailed assessment of functionally relevant characters and supports interpretation of a benthic lifestyle on soft, low-energy substrates. A conservative concept of the family and a combination of body, pleon-pleotelson and antennular features justifies the placement of *Cicero* n. gen. within this extinct sphaeromatidean lineage. Within this framework, the occurrence *C. spectabilis* n. gen. n. sp. in a stratified lacustrine system of the Araripe Basin, together with other Mesozoic archaeoniscid-like records, suggest that some lineages currently assigned to Archaeoniscidae were able to colonize a range of non-marine environments during the Early Cretaceous, while also underscoring the need for a comprehensive phylogenetic reassessment of the group.

## Introduction

The Archaeoniscidae^1^ comprises an extinct lineage of dorsoventrally flattened isopod crustaceans known primarily from the Mesozoic fossil record. Characterized by a broad pleotelson, compact cephalon, dorsoventrally flattened body and prominent antennae^2^, members of this family are typically interpreted as benthic organisms adapted to shallow marine or marginal lacustrine environments^3^. The family currently includes three genera: *Archaeoniscus*^4^, with a wide paleogeographic distribution from the Jurassic to Cretaceous of Europe and Asia^5^, * Ferreniscus*^6^, from the Upper Jurassic of Spain, and *Codoisopus*^7^, from the Cretaceous Codó Formation in northern Brazil, hitherto representing the only known occurrence of the family in Gondwana. These genera highlight the morphological conservatism and potential ecological versatility of archaeoniscids, though their precise phylogenetic position within Isopoda remains unclear^3^.

The Crato Formation, part of the Santana Group within the Araripe Basin of northeastern Brazil, represents one of the most significant Konservat-Lagerstätten of the Early Cretaceous, particularly the Aptian stage^8,9^. Deposited in a stratified lacustrine system under semi-arid climatic conditions, the Crato limestones are renowned for their fine lamination and outstanding richness of fossils, including exceptionally preserved insects, arachnids, decapod crustaceans, fishes, pterosaurs, and plants (e.g.,^10–12^). Considering the overall paleobiodiversity of Crato, it is almost surprising that not a single member of Isopoda, one of the morphologically and ecologically most diversified peracarid orders^13,14^, has been previously recorded from this formation, with the exception of the recently documented parasite-host interaction between epicaridean isopods and marine shrimps^15^. Generally, Isopoda is represented by very few fossil records in Brazil, where this group has been historically underrepresented or misclassified^7,16^.

Herein, we describe a new genus and species of Archaeoniscidae based on a single specimen from the Nova Olinda Member of the Crato Formation, representing the first occurrence of the family in this paleontological unit. Our primary aim is to document the morphology of *Cicero spectabilis* n. gen. n. sp. and to discuss its most plausible systematic placement within Isopoda, rather than to provide a comprehensive taxonomic revision of Archaeoniscidae. The exceptional preservation of the holotype allows not only detailed morphological comparisons between the new genus and previously described archaeoniscid genera, but also tentative interpretations of the paleobiology of the new species.

### Geological setting

The Crato Formation, located in the Araripe Basin of southern Ceará, northeastern Brazil, represents a geologically and paleontologically significant unit of the Early Cretaceous, particularly the Aptian stage (122–113 mya)^17^. The Araripe Basin formed as a result of rifting associated with the initial separation of South America and Africa during the breakup of Gondwana^18^. The Crato Formation, part of the Santana Group’s post-rift sequence, is an important Konservat-Lagerstätte well-known for its fossil deposits, offering exceptional preservation of both flora and fauna^9^.

Stratigraphically, the Crato Formation lies above the Barbalha Formation and below the Ipubi Formation. It is subdivided into several members, with the Nova Olinda Member being the lowest and most fossiliferous. This unit is characterized by rhythmic pale to dark laminations in micritic limestone, indicating specific paleoenvironmental conditions, such as anoxic and hypersaline bottom waters, limited siliciclastic input, and a restricted continental freshwater system gradually transitioning into a more open lacustrine setting^8,10^. Lithologically, the formation consists of laminated limestones interbedded with marls, shales, clays, and occasional sandstones^19^.

The laminated limestones, particularly within the Nova Olinda Member, formed primarily through authigenic calcite precipitation, likely mediated by phytoplankton and picoplankton activity, rather than by benthic microbial mats^8^. These sediments show strong facies differentiation, with pale yellow limestones richer in fossil content and suggesting shorter transport distances for insects, for instance, compared to darker layers^20^. A laterally continuous sedimentary layer within the Crato Member has been linked to region-wide microbialite formation, interpreted as autochthonous, biologically induced mineralization during a specific paleoenvironmental event^12^.

Paleoenvironmental interpretations support a lacustrine system sensitive to climatic and hydrological fluctuations. The Crato Formation’s microbial limestones function as valuable proxies for environmental reconstruction, capturing variations in salinity, water chemistry, and sedimentation patterns^12^. A notable feature within this formation, the Caldas Bed, records a short-term freshening event indicated by changes in bathymetry and sedimentological characteristics, further affirming the predominantly freshwater nature of the depositional environment, despite occasional marine mollusk occurrences in older literature^21^. Thus, the Crato Formation of the Araripe Basin offers an integrated record of Early Cretaceous sedimentary dynamics, paleoclimate, and biotic evolution. Its finely laminated limestones, distinctive stratigraphy, and remarkable fossil content make it a globally significant reference point for understanding continental lacustrine systems during the early stages of the South Atlantic rifting.

## Material and methods

Descriptions, illustrations, and photographs were conducted using a Nikon SMZ 745 T stereomicroscope equipped with a camera lucida and a Leica EZ4 W stereomicroscope, both fitted with digital imaging systems. Whole specimen photographs were taken with a Canon G10 digital camera. The holotype was examined without coating and ultraviolet (UV) light was employed to enhance the visibility of certain anatomical structures.

Terminology used in the morphological descriptions follows Park et al.^3^ and Jones et al.^2^. All measurements are reported in millimeters (mm), and the following abbreviations are used: L/W, length-to-width ratio. † indicates exclusively fossil taxa. The life reconstruction in Fig. 3 necessarily depicts the animal in dorsal view and included inferred dorsal features and coloration; these aspects are hypothetical and constrained primarily by the ventral morphology preserved in MPSC 9701 and by comparison with extant sphaeromatidean isopods.

The specimen was found among a small collection of fossil arthropods that had been donated to the Illinois Center for Paleontology at the University of Illinois by an anonymous donor. It was subsequently repatriated to Brazil and is now housed in the collection of the Museu de Paleontologia Plácido Cidade Nuvens (MPPCN) under the registration number MPSC 9701. Permission to access, analyze, and publish data on these materials was granted by the institution.

## Results

### Systematic paleontology

**Order** Isopoda^22^

**Suborder** Sphaeromatidea^23^

**Family** Archaeoniscidae†^1^

**Diagnosis**. Isopods with broad, ovate to sub-ovate, dorsoventrally flattened body; cephalon compact, at least partly set into pereonite 1; lateral margins of pereon defined by broad coxal plates; pleon as wide as pereon, with four large free pleonites and a reduced fifth; pleotelson large, forming a broad terminal shield; uropods inserted ventrolaterally on pleotelson, with rami moderately to well developed.

**Remarks**. This operational and broad diagnosis is a synthesis of observations made by Calzada & Urquiola^6^, Calzada et al.^24^ and Park et al.^3^, and is intended purely as a working concept and not a final delineation, pending a comprehensive revision of the group based on reassessment of all type material.

Included genera. *Archaeoniscus*†^4^ (type genus),* Cicero*† n. gen., *Codoisopus*†^7^, * Ferreniscus*†^6^.

*Cicero* n. gen.

LSID: https://zoobank.org/541C831F-9A6D-4EB3-ADD6-BE73EF173D7F

**Type species**.— *Cicero spectabilis* n. gen. n. sp*.* by present designation and monotypy, gender masculine.

**Diagnosis**.— Overall body oval in shape. Frontal lamina longer than wide, broadened anteriorly, with small median point. Uropods with exopod and endopod clearly separated, forming together with pleotelson broad, fan-like caudal structure; uropods slightly overreaching posterior margin of pleotelson. Pleotelson rounded-subrectangular, bearing multiple small, spiniform setae.

**Etymology**.— The new genus is named in honor of Padre Cícero Romão Batista (“Padre Cícero”), a prominent religious leader who was born and established his legacy in the cities of Crato and Juazeiro do Norte, both in the Araripe Basin, northeastern Brazil. Gender masculine.

***Remarks***.— The inclusion of *Cicero* n. gen. in Archaeoniscidae is supported by several characters, such as (1) broad, oval, moderately vaulted body; (2) similar pereonites, except for the first; (3) pleon as wide as pereon; (4) pleonites I–V free, subequal; and (5) large pleotelson (sensu^6^). In addition, the antennular morphology diverges from the condition typically observed in members of Sphaeromatidae. In most sphaeromatid genera, the antennular articles follow a consistent pattern: the first article is the longest and broadest; the second article is the shortest but nearly as wide as the first; and the third article is slightly longer but significantly narrower than the preceding ones^25^. In contrast, *Cicero* n. gen. displays an antennular configuration characterized by a gradual increase in length across the articles, with the first article being the shortest, followed by the slightly longer second article, while the third article is both longer and narrower, thus differing from the compact, broadened antennules typical for sphaeromatids. The elongate, distally tapering antennules of *Cicero* n. gen. are more similar to the antennular configuration seen in other archaeoniscid taxa, thereby reinforcing the systematic placement of the new genus within Archaeoniscidae rather than Sphaeromatidae. None of the individual traits used here to place *Cicero* n. gen. within Archaeoniscidae is in itself unequivocally apomorphic at family level; instead, the assignment reflects a combination of characters consistent with the operational diagnosis of the group (see above) and the absence of clear affinities with any extant sphaeromatidean family.

**Fig. 1:**
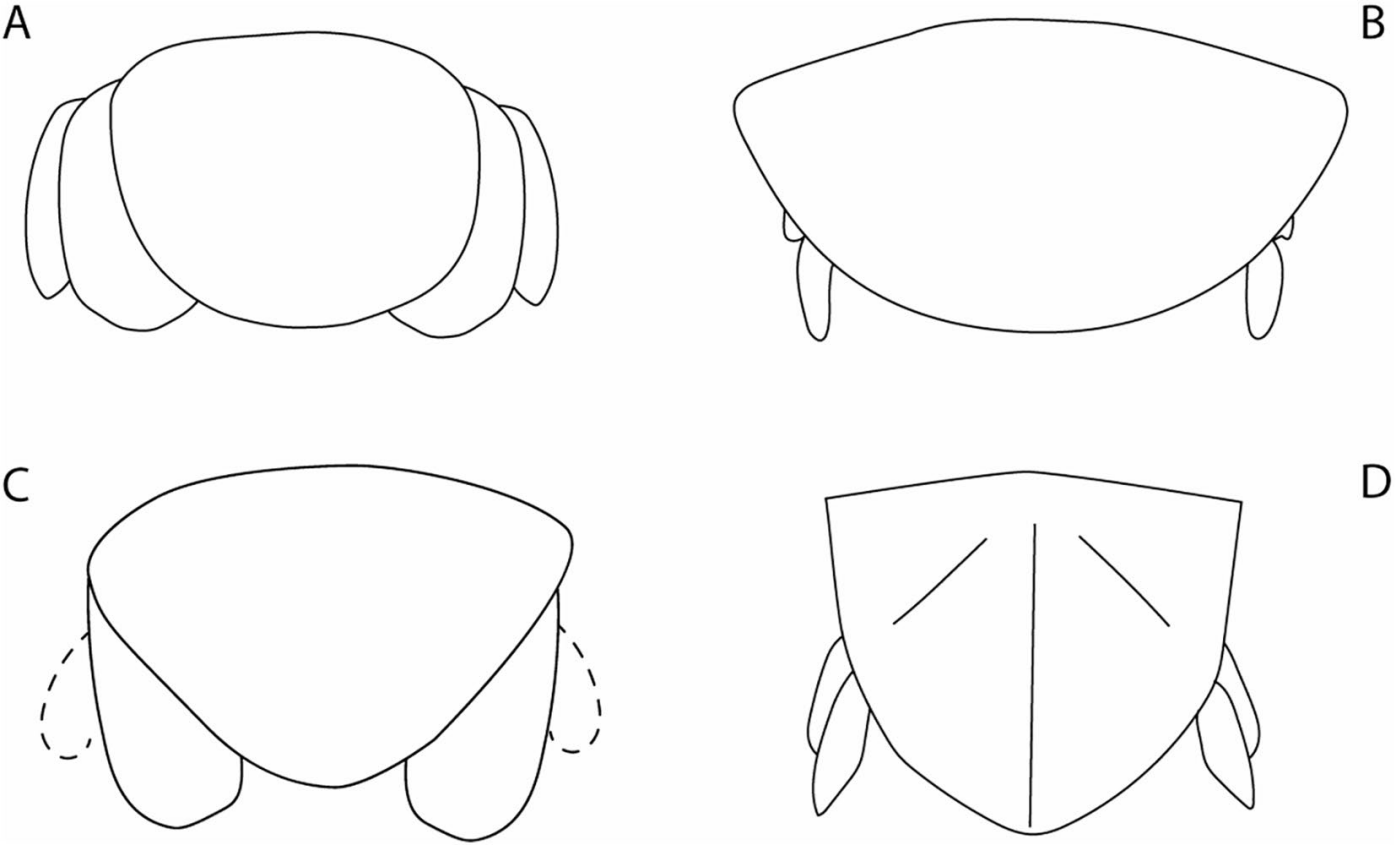
Non-scaled morphological features of the posterior portion of the body in Archaeoniscidae. A, *Cicero* n. gen. (this study, based on MPSC 9701). B, *Archaeoniscus* (outline based on^3^). C, *Codoisopus* (outline based on^7^)*.* D, *Ferreniscus* (outline based on^24^). All silhouettes are interpretative and reflect the quality of preservation and published illustrations for each taxon.

*Cicero* n. gen. can be easily distinguished from other members of the Archaeoniscidae using several morphological features of the posterior portion of the body. Most notably, *Cicero* n. gen. exhibits biramous uropods that are clearly subdivided into an exopod and an endopod, which together with the pleotelson form a broad, fan-like caudal structure (Fig. 1A). This contrasts sharply with *Archaeoniscus*, in which the uropods are slender, rod-like, and only weakly differentiated into rami (Fig. 1B). This pronounced difference in the uropod structure suggests different functional morphology and possibly divergent ecological adaptations. *Cicero* n. gen. superficially resembles *Codoisopus*, particularly in the fan-like uropodal arrangement. However, the new genus is readily distinguishable from *Codoisopus* by the shape of the pleotelson, which is rounded-rectangular, with a nearly straight posterior margin furnished with multiple spiniform setae (Fig. 1A) *versus* a more angular, rounded-triangular pleotelson, with an unarmed posterior margin (although it may reflect the imperfect preservation of the presently available specimens) in *Codoisopus* (Fig. 1C). Finally, *Cicero* n. gen. markedly differs from *Ferreniscus*, in which the body is more cylindrical in outline(^24^: 45, Fig. 1b) instead of being broadly ovate as in *Cicero* n. gen. Furthermore, the pleotelson in *Ferreniscus* is ovately triangular and has a prominent dorsal carina (Fig. 1D), two characteristics not observed in *Cicero* n. gen. (Fig. 1A). We believe that the differences across multiple key taxonomic characters, viz. general body shape and morphology of the uropods + pleotelson (caudal appendage) are significant enough to support the establishment of *Cicero* n. gen. as a new genus within Archaeoniscidae.

In addition to morphological separation between *Cicero* n. gen. and *Codoisopus*, the distinction between these two genera is supported by geological and paleoenvironmental differences between the Crato and Codó formations. The Codó Formation, located in the Parnaíba Basin, presumably represents a marginal marine or restricted lagoonal environment, influenced by episodic marine incursions during the Early Cretaceous^7^. In contrast, the Crato Formation within the Araripe Basin is interpreted as a stratified hypersaline to freshwater lacustrine system deposited under semi-arid climatic conditions^8,9,21^. These distinct depositional settings may have imposed different selective pressures, leading to divergent morphological adaptations, particularly in body shape and caudal structures. The more angular pleotelson of *Codoisopus* may reflect adaptation to a more dynamic, possibly shallow marine, marginal environment, whereas the rounded pleotelson with spine-like setae in *Cicero* n. gen. could be related to a more stable, sediment-rich lacustrine habitat.

*Cicero spectabilis* n. sp*.*

LSID: https://zoobank.org/9DF28C31-99E5-4C41-99EC-473390D02C24

Figures 1A, 2, 3

**Fig. 2:**
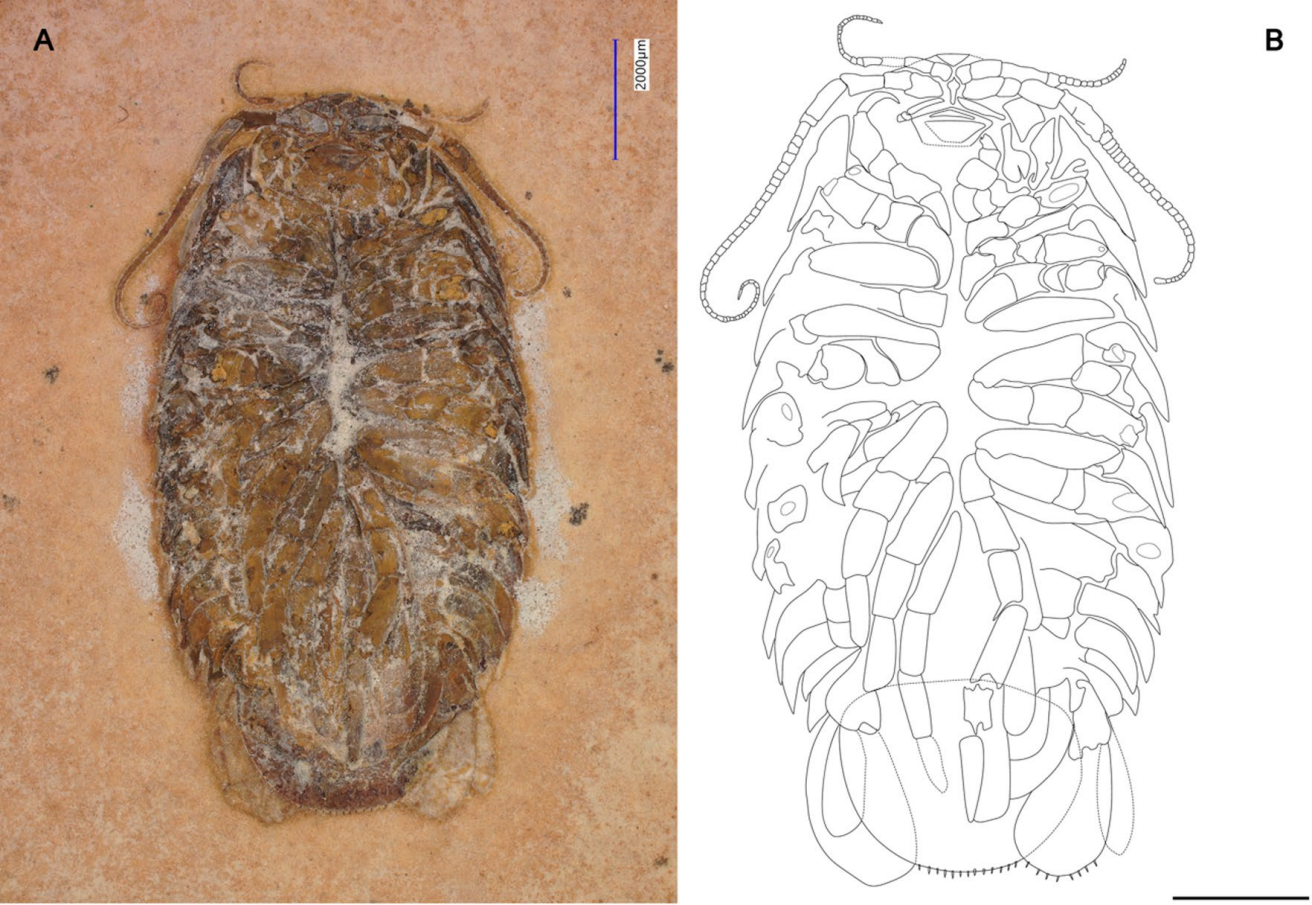
Holotype of *Cicero spectabilis* n. gen. n. sp*.* from the Aptian Nova Olinda Member of the Crato Formation, Araripe Basin, Brazil (MPSC 9701). A, Ventral view of the specimen preserved in laminated limestone. B, Interpretative line drawing highlighting cephalic appendages, pereopods, pleon, pleotelson, and uropods. Scale bars: 2 mm.

**Fig. 3:**
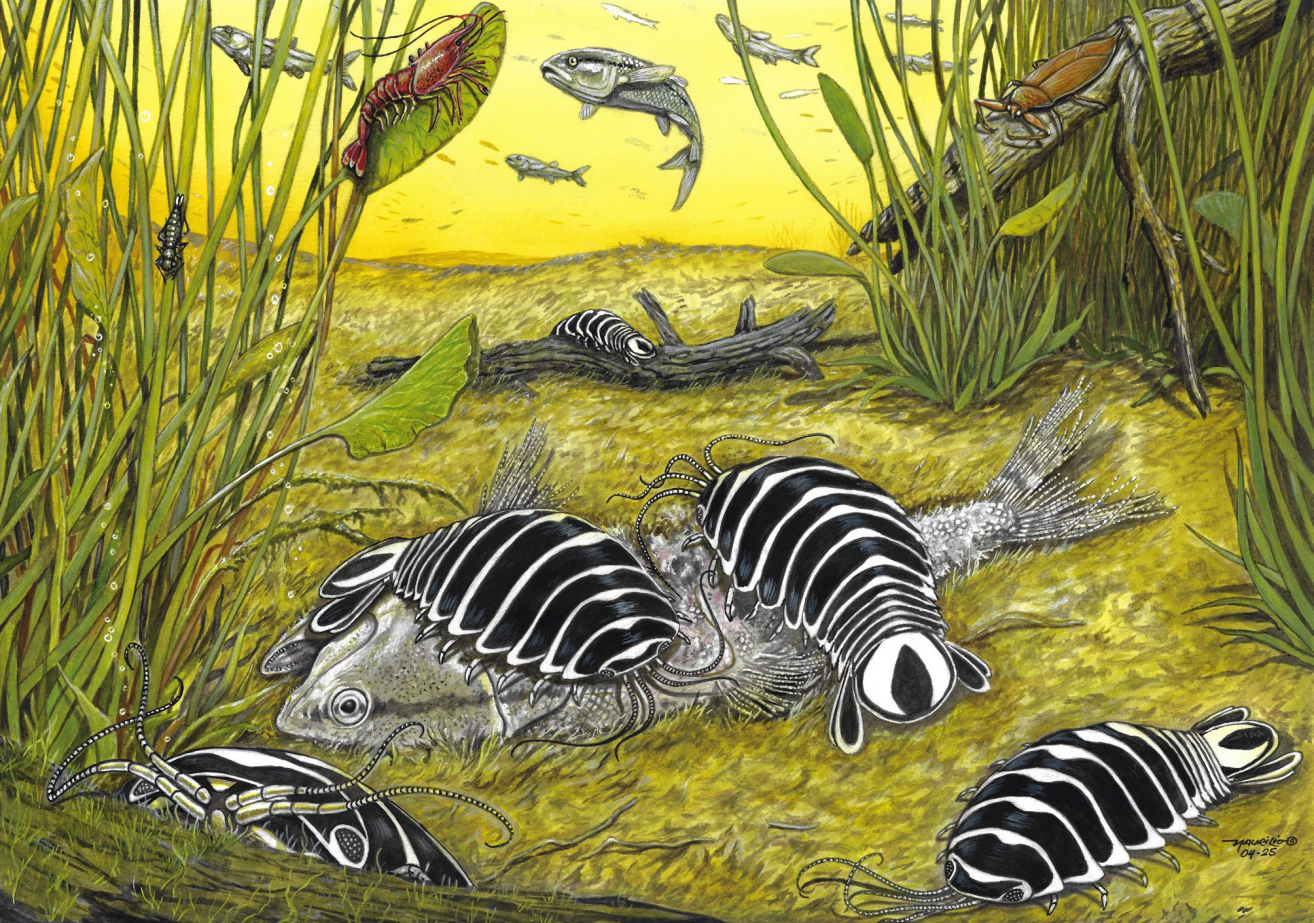
Life reconstruction of *Cicero spectabilis* n. gen. n. sp*.* in the Aptian lacustrine environment of the Crato Formation, Araripe Basin, Brazil, showing several individuals scavenging on a dead fish (*Dastilbe*) on the lake floor, surrounded by aquatic vegetation (*Notocyamus hydrophobus*), fishes and arthropods. Coloration and most dorsal features are conjectural and based on extant sphaeromatidean isopods. Illustration by Maurílio Oliveira (paleoartist, MN/UFRJ).

**Holotype**.— MPSC 9701 (Fig. 2).

**Type locality**.— Exact locality unknown; one of numerous laminated limestone quarries in the region of the municipality of Nova Olinda; Ceará, Brazil.

**Stratigraphic unit**.— Nova Olinda Member, Crato Formation, Santana Group.

**Type age**.— Early Cretaceous, Aptian (122–113 mya).

**Diagnosis**.— See under genus.

**Description**.— Overall body form broad, oval in shape; total length 12.0 mm; width 6.4 mm.

Head trapezoidal in ventral view, apparently set into pereonite 1; frontal margin rounded. Eyes not preserved. Frontal lamina longer than wide, spatulate shaped anteriorly. Clypeus and labrum distinct; clypeus rounded triangular, narrow than labrum; labrum trapezoidal, almost twice as longer than clypeus.

Pereon 0.57 times longer than total body length, 3.5 times longer than pleon; widest at the level of the pereonite 4 and 5. Pereonite 1 longer than other pereonites; anterior margin depressed to embrace head; posterolateral margin of coxal plate 1 strongly angular. Pereonites 2–6 apparently of similar shape, with posterolateral margin of coxal plates 2–6 angular. Pereonite 7 slightly shorter than pereonites 2–6; posterolateral margin angular, but angles not as pronounced as those of pereonites 1–6.

Pleon 0.17 times longer than total body length; 0.8 times longer than pleotelson. Pleonites 1–4 with posterolateral corner angular. Pleonites 2–4 subequal in length and width. Pleonite 1 slightly reduced in width and length compared to pleonites 2–4. Pleonite 5 not discernible. Pleopod insertions not discernible.

Pleotelson 0.2 times longer than total body length. Pleotelson posteriorly semicircular, broad, with row of setae on posterior margin; lateral margin rounded, smooth. Pleotelson and uropods together forming broad, fan-like caudal structure.

Antennule inserting to anterolateral margin of frontal lamina. Antennular peduncle with 3 articles; article 1 shortest; subsequent articles increasing in size gradually. Antennular flagellum with at least 21 subdivisions.

Antenna inserting to lateral margin of frontal lamina; twice as long as antennule. Antennal peduncle with 4 articles; articles 1 and 2 similar in size, shorter than articles 3 and 4; articles increasing in size gradually. Antennal flagellum with 47 subdivisions.

Mouthparts not well preserved. Left maxilliped poorly preserved, consisting of 5 articles, apparently with endite on article 1.

All pereopods functionally developed as ambulatory legs. Right pereopod 1 complete; basis 3 times as long as wide; as long as combined length of ischium, merus, and carpus; propodus and dactylus similar in size; dactylus 3.5 times as long as wide. Almost all remaining pereopods not well preserved. Pereopods 2–4 similar in size, with basis as long as combined length of ischium and merus; dactylus not preserved. Pereopod 5–7 similar in size, longer than pereopods 1–4; ischium, merus, carpus, propodus, and dactylus similar in size.

Uropodal insertion to pleotelson ventrolateral; uropod slightly surpassing distal margin of pleotelson. Protopod longer than wide; about 0.4 uropod length, not exceeding distal margin of pleotelson. Endopod broad, extending beyond pleotelson. Exopod shorter than endopod, not reaching distal margin of pleotelson.

**Etymology**.— The specific epithet *spectabilis* (Latin for notable, outstanding, spectacular) refers to the remarkable quality of preservation of the holotype specimen. Used as an adjective.

## Discussion

### Systematic affinities

The discovery of *Cicero spectabilis* n. gen. n. sp. in the Aptian Crato Formation adds an important new datapoint to the sparse fossil record of isopods in South America and, more specifically, to our understanding of Archaeoniscidae in Gondwanan settings^7,26,27^. Prior to this study, only *Codoisopus brejensis* from the Aptian Codó Formation and the problematic *Unusuropode castroi* from the Turonian Apodi Group were known from the Cretaceous of Brazil, with the latter taxon assigned to Sphaeromatidae and later reported from North Africa^7,26,27^. The unambiguous archaeoniscid affinities of *Cicero* n. gen. as defined under the current, preliminary concept of the family, extend the record of archaeoniscid-like isopods within Gondwana from marginal‑marine or restricted lagoonal deposits of the Parnaíba Basin to a fully lacustrine system in the Araripe Basin and complement the freshwater record of *Archaeoniscus coreaensis* from the Jinju Formation in East Asia^3^.

Collectively, the known occurrences of *Archaeoniscus* in Upper Jurassic and Lower Cretaceous shallow-marine to lagoonal deposits in Europe, Mexico and Egypt^3,28–31^, and of *Cicero* n. gen. in the lacustrine Crato Formation of Brazil suggest that some lineages currently assigned to Archaeoniscidae occupied a range of non‑marine environments, from marginal-marine or restricted lagoonal settings to inland lacustrine systems.

Whether this breadth reflects true ecological versality within a single clade or artificial inclusion of several lineages with partly distinct histories under a provisional (and possibly non-monophyletic, see below) family concept, remains to be tested by future phylogenetic analyses. In contrast to many crustacean groups (e.g. Decapoda), for which strongly habitat-restrict lineages and tight environmental partitioning are well documented, the apparent spread of archaeoniscid-like isopods across different near-shore and continental water bodies may partly reflect the still fragmentary and taxonomically unresolved fossil record of the group^3,28^.

The paleoenvironmental context of the Crato and Jinju formations provides an important framework for interpreting the ecology of the archaeoniscid-type isopods. The Nova Olinda Member of the Crato Formation represents a stratified lacustrine system developed in an interior basin during the early stages of South Atlantic rifting^8,9,18^. Finely laminated micritic limestones, with alternation of pale and dark laminae, indicate low‑energy deposition, episodic anoxia and variable salinity under semi‑arid climatic conditions^8,10,12,21^. Insects, arachnids, decapods, fishes and plants are often preserved with exceptional fidelity, testifying to rapid burial and minimal transport in a water column characterized by strong chemical and redox stratification^9,17,20^. In contrast, the Jinju Formation records a fluviolacustrine system in the Gyeongsang back‑arc basin, with sedimentological and fossil evidence (e.g., freshwater mollusks, ostracods, “conchostracans” and aquatic insects) pointing to predominantly freshwater conditions^32–35^.

*Archaeoniscus coreaensis* represents the first unequivocal occurrence of the genus in an exclusively freshwater setting, demonstrating that *Archaeoniscus* successfully invaded inland lacustrine habitats in East Asia^3^. *Cicero spectabilis* n. gen. n. sp. preserved in laminated Crato limestones, likely occupied a similar benthic niche in soft, low‑energy substrates, but within a stratified lake that experienced periodic fluctuations in salinity and oxygen availability^8,21^. The combination of these two records suggests that at least some archaeoniscid-like lineages tolerated variable salinity and redox conditions and were able to inhabit both marginal-marine and lacustrine settings. Given the current uncertainty surrounding the limits and internal relationships of Archaeoniscidae, we regard this ecological plasticity as a working hypothesis rather than a firmly established family-level attribute.

### Paleobiology

The exceptional preservation of *Cicero spectabilis* n. gen. n. sp. permits a detailed assessment of functionally relevant characters and supports a benthic lifestyle on soft, low-energy substrates, consistent with previous inferences for *Archaeoniscus coreaensis*^3^. In overall body shape and inferred mode of life, archaeoniscid-like isopods are broadly comparable to dorsoventrally flattened, epibenthic, extant sphaeromatidean taxa (e.g., some Sphaeromatidae and Serolidae) that inhabit shallow-marine, marginal and, more rarely, brackish to freshwater environments^25,36,37^. The broad, ovate body of *C. spectabilis* n. gen. n. sp. in conjunction with relatively simple ambulatory pereopods and a fan‑like caudal region formed by the pleotelson and biramous uropods, is well suited for locomotion and stabilization on soft lacustrine substrates. The uropodal exopod and endopod are robust and only slightly overreach the pleotelson, suggesting a role in steering, substrate interaction and perhaps limited digging rather than in specialized attachment or swimming.

There is no evidence of hook‑like modifications of the anterior pereopods, nor of any other morphological traits that would support an ectoparasitic lifestyle analogous to that of cymothoid isopods on fishes^3,28,38^. This is congruent with the conclusions of Park et al.^3^, who rejected the ectoparasite hypothesis for *A. coreaensis* based on the largely unmodified ambulatory limbs and the absence of direct fossil associations with fish hosts, and instead proposed a benthic mode of life comparable to that of extant serolids and other sphaeromatidean isopods^36,39^. In this context, *Cicero spectabilis* n. gen. n. sp. reinforces the view that archaeoniscids were primarily benthic organisms occupying soft‑bottom microhabitats in shallow lacustrine and marginal‑marine settings, rather than highly specialized ectoparasites.

Preservational aspects of both the Crato and Jinju material also are relevant for the interpretation of putative phylogenetic characters in Archaeoniscidae. Park et al.^3^ demonstrated that the axial posterior ridge previously interpreted as a pleonal brood pouch in some species of *Archaeoniscus*^28,31^ instead corresponds to the preserved hindgut, a conclusion supported by specimens of *A. coreaensis*, in which remnants of the foregut and hindgut can be traced. This reinterpretation removes a supposed family‑level synapomorphy and aligns Archaeoniscidae with the peracarid pattern of a thoracic ventral brood pouch formed by oostegites in females, rather than a unique pleonal reproductive structure^40^. The Crato specimen of *Cicero spectabilis* n. gen. n. sp. does not show any axial structure that could be reasonably interpreted as a brood chamber, and its preservation is consistent with a typical soft‑tissue decay and mineralization pathway in laminated limestones^8,20^. The convergence of taphonomic and anatomical evidence from these two formations therefore underscores the risk of over‑interpreting taphonomically enhanced axial ridges as primary reproductive characters, and cautions against using such features to diagnose higher‑level clades within Isopoda.

Within this paleobiological and taphonomic framework, the new Brazilian fossil contributes directly to the long‑standing debate on the systematic position of Archaeoniscidae. *Archaeoniscus* has been variably allied with Oniscidea, Flabellifera and Sphaeromatidea, and comparisons have been drawn to both Sphaeromatidae and Cymothoidae based on superficial similarities in body outline and presence of presumed brood pouches^3,28,31,41–44^. The detailed redescription of *A. coreaensis* by Park et al.^3^, based on more than 100 specimens, led to the recognition of (1) oval, dorsoventrally compressed body with the cephalon set into the first pereonite, (2) broad overlapping coxal plates forming the lateral margins of the pereon, (3) pleon as wide as pereon, with four large pleonites and a reduced fifth, (4) broad semicircular pleotelson lacking marginal spines, (5) elongate multiarticulate antennules, and (6) narrow, spine‑like uropodal rami. These traits are broadly consistent with a position within Sphaeromatidea, under Sphaeromatoidea^23,31,36^, but do not match the characteristic morphologies of Oniscidea or Sphaeromatidae, nor do they support close affinities with Cymothoida^25,37^.

While *Cicero spectabilis* n. gen. n. sp. exhibits key archaeoniscid features, such as a broad, flattened body, pleon as wide as pereon and a large pleotelson, it also adds new information on the diversity of caudal and cephalic appendages within the family. Most notably, *Cicero* n. gen. has biramous uropods with well‑differentiated exopod and endopod, which together with a rounded‑subrectangular pleotelson bearing a row of posterior setae form a broad fan‑like caudal structure, differing markedly from the narrow, rod‑like uropods and semicircular pleotelson of *Archaeoniscus*^3^. The antennular peduncle of *Cicero* n. gen. with articles increasing gradually in length and a distally tapering configuration, also contrasts with the compact, broadened antennules typical of many sphaeromatids and more closely resembles the elongate, subdivided antennules of *Archaeoniscus*^3,25^.

Even though the above-listed characters support the inclusion of *Cicero* n. gen. in Archaeoniscidae and reinforce the distinction between archaeoniscids and Sphaeromatidae, we recognize that the family, as currently delimited, is defined by a relatively broad set of mostly plesiomorphic characters and may not represent a morphologically homogeneous group and/or monophyletic clade within Sphaeromatoidea^3,6,7,24^. For instance, Fig. 1 illustrates marked differences in the outline of the pleotelson and uropodal morphology among the four genera currently included in this family. However, we are aware that part of this variation may reflect preservation-related bias and use of simplified interpretative drawings for *Ferreniscus* and *Codoisopus*. Without access to the type material of these taxa, any interpretations of their morphological details must be regarded as provisional.

### Paleogeography

From a paleogeographical perspective, the combined occurrence of *Archaeoniscus coreaensis* in the Jinju Formation and *Cicero spectabilis* n. gen. n. sp. in the Crato Formation has important implications for Early Cretaceous patterns of crustacean diversification. *Archaeoniscus* was previously known mainly from Upper Jurassic to Lower Cretaceous deposits of Europe, Mexico and Egypt, occupying shallow marine, lagoonal and marginal environments along the margins of the central Atlantic and western Tethys^28–31,45^. The Jinju record extended this distribution into East Asia and demonstrated successful colonization of inland freshwater systems in a back‑arc basin^3^.

*Cicero* n. gen. now shows that archaeoniscids were also present in interior basins of northeastern Brazil during the Aptian, at a time when these basins were influenced by episodic marine incursions but dominated by lacustrine conditions^8,18,21,46^. This pattern is consistent with the idea that some archaeoniscid-like lineages participated in the broader radiation of peracarid crustaceans associated with the fragmentation of Gondwana and the development of complex networks of marginal‑marine and continental basins^5,47^. However, with only a handful of well-documented species and without a formal phylogenetic analysis that integrates all known taxa, the timing and extend of any such radiation remain speculative.

The new record of Archaeoniscidae from the Crato Formation also fits within a broader Tethyan framework that has been invoked for other isopod lineages. Direct evidence of epicaridean parasitism on decapods, including the earliest bopyrid–shrimp association from the Aptian Romualdo Formation of the Araripe Basin, suggests that the western Tethys acted as a major center of origin and dispersal for parasitic isopods, with Jurassic and Early Cretaceous epicaridean swellings (*Kanthyloma crusta*), which have been attributed to bopyrids and other epicarideans concentrated in European Tethyan basins^15,48^. In a similar way, most Jurassic and Lower Cretaceous records of *Archaeoniscus* are restricted to shallow-marine and lagoonal deposits along the margins of the central Atlantic and western Tethys, indicating that the family initially diversified in Tethyan and para-Tethyan settings before expanding into continental interiors^3,28–31^.

The occurrences of *Archaeoniscus coreaensis* in the non-marine Jinju Formation of Korea and *Cicero spectabilis* n. gen. n. sp. in the lacustrine Crato Formation of Brazil can thus be interpreted as part of a second phase of dispersal, in which archaeoniscid lineages that had evolved along Tethyan margins tracked newly formed back-arc and rift-related basins during the fragmentation of Gondwana and the progressive narrowing of the Tethys seaway^18,19^.

In light of these morphological, ecological and biogeographical considerations, we adopt a deliberately conservative stance regarding the suprageneric placement of Archaeoniscidae. The available evidence strongly supports assignment of the family to Isopoda, suborder Sphaeromatidea, with general affinities to Sphaeromatoidea, on the basis of body outline, pleon and pleotelson development, and uropodal configuration^3,23,31,36^. However, the specific combination of characters exhibited by *Archaeoniscus* and *Cicero* n. gen. including a pleon as wide as pereon, a large pleotelson, ventrolateral uropods, elongate antennules and simple ambulatory pereopods, does not allow a firm placement of these taxa in any extant sphaeromatidean family^25,37^.

We therefore regard Archaeoniscidae as an extinct, morphologically reasonably distinctive lineage within Sphaeromatidea. The relationships of this family to other sphaeromatidean lineages remain unresolved, however, pending a comprehensive phylogenetic analysis integrating both fossil and living taxa. In this context, *Cicero spectabilis* n. gen. n. sp. not only expands the known range and ecological diversity of Archaeoniscidae, but also highlights the importance of Konservat‑Lagerstätten, such as the Crato Formation, for refining higher‑level systematic hypotheses in Isopoda.

## Data Availability

All data generated and analyzed during this study are included in this published article. Additional high-resolution images and measurement data are available from the corresponding author on request. The holotype specimen (MPSC 9701) is deposited in the paleontological collection of the Museu de Paleontologia Plácido Cidade Nuvens (MPPCN), Santana do Cariri, Ceará, Brazil.
